# Mass-independent oxygen isotope fractionation driven by energetic-particle irradiation of icy solids

**DOI:** 10.1073/pnas.2610806123

**Published:** 2026-10-21

**Authors:** Gerardo Dominguez, Caroline Caplan, Lauren Tafla, Joshua Lucas, Ming-Chang Liu, Kevin McKeegan, Jeff Gillis

**Affiliations:** ^a^ Department of Physics, California State University, San Marcos, CA 92096-0001; ^b^ Department of Earth, Planetary, and Space Sciences, University of California, Los Angeles, CA 90095-1567; ^c^ Department of Physics, Washington University of St. Louis, St. Louis, MO 63130

**Keywords:** mass-independent isotope fractionation, oxygen isotopes, radiation chemistry, interstellar dust, solar system history

## Abstract

Mass-independent isotope fractionation (MIF) of oxygen provides key insights into atmospheric chemistry and the origin of isotopic anomalies in planetary materials. To date, MIF has been primarily associated with gas-phase processes involving symmetry-dependent reaction pathways, most notably ozone formation. Whether analogous effects can arise in condensed phases or at interfaces remains an open question. Here we report laboratory experiments demonstrating that energetic particle irradiation of icy oxide surfaces produces mass-independent oxygen isotope fractionation at temperatures as low as 10 K. Electron irradiation of SiO_2_ and Al_2_O_3_ substrates in the presence of H_2_O ice results in measurable shifts in Δ^17^O in the underlying solids. The resulting three-isotope relationships include slope-1-like behavior in SiO_2_ and Al_2_O_3_ and distinct, substrate-dependent trends in Al_2_O_3_, indicating that irradiation-induced defect formation and interfacial chemistry govern the isotope effects. The magnitude of the observed anomalies demonstrates that irradiation-driven processes can generate substantial oxygen isotope heterogeneity in condensed phases. These results identify a previously unrecognized class of MIF and provide a quantitative framework for interpreting oxygen isotope signatures in radiation-processed environments, including interstellar grains and early solar system materials.

## Significance

Mass-independent oxygen isotope effects are fundamental to understanding atmospheric chemistry and planetary formation, yet they have been largely confined to gas-phase processes such as ozone formation. We show that irradiation of icy solid surfaces produces large mass-independent oxygen isotope effects at cryogenic temperatures. The isotope signatures depend on the underlying material, revealing a new, substrate-controlled mechanism operating in condensed phases. This work expands the known regimes of mass-independent chemistry and suggests that radiation-driven surface processes may contribute to isotopic signatures in planetary materials and interstellar dust.

Mass-independent oxygen isotope fractionation (MIF) is a defining characteristic of planetary materials and a central constraint on models of solar system formation (1, 2). The standard model of planetary system formation, supported by astronomical observations, describes the gravitational collapse of gas and dust within molecular clouds, followed by the growth of solids through collisional aggregation into planetesimals (1). While this framework provides important context, the most stringent constraints on the solar system’s formation history are derived from the isotopic composition of major planet-forming elements. Despite extensive study, the physicochemical mechanisms capable of generating MIF in solids remain incompletely understood. In particular, it is unclear whether irradiation-driven chemical processes acting on cold, icy solids can produce MIF under astrophysically relevant conditions. Here, we report laboratory evidence that energetic particle irradiation of icy oxide surfaces produces mass-independent oxygen isotope fractionation in solids at cryogenic temperatures, identifying a previously unrecognized mechanism for generating MIF in condensed phases.

**Fig. 1.**
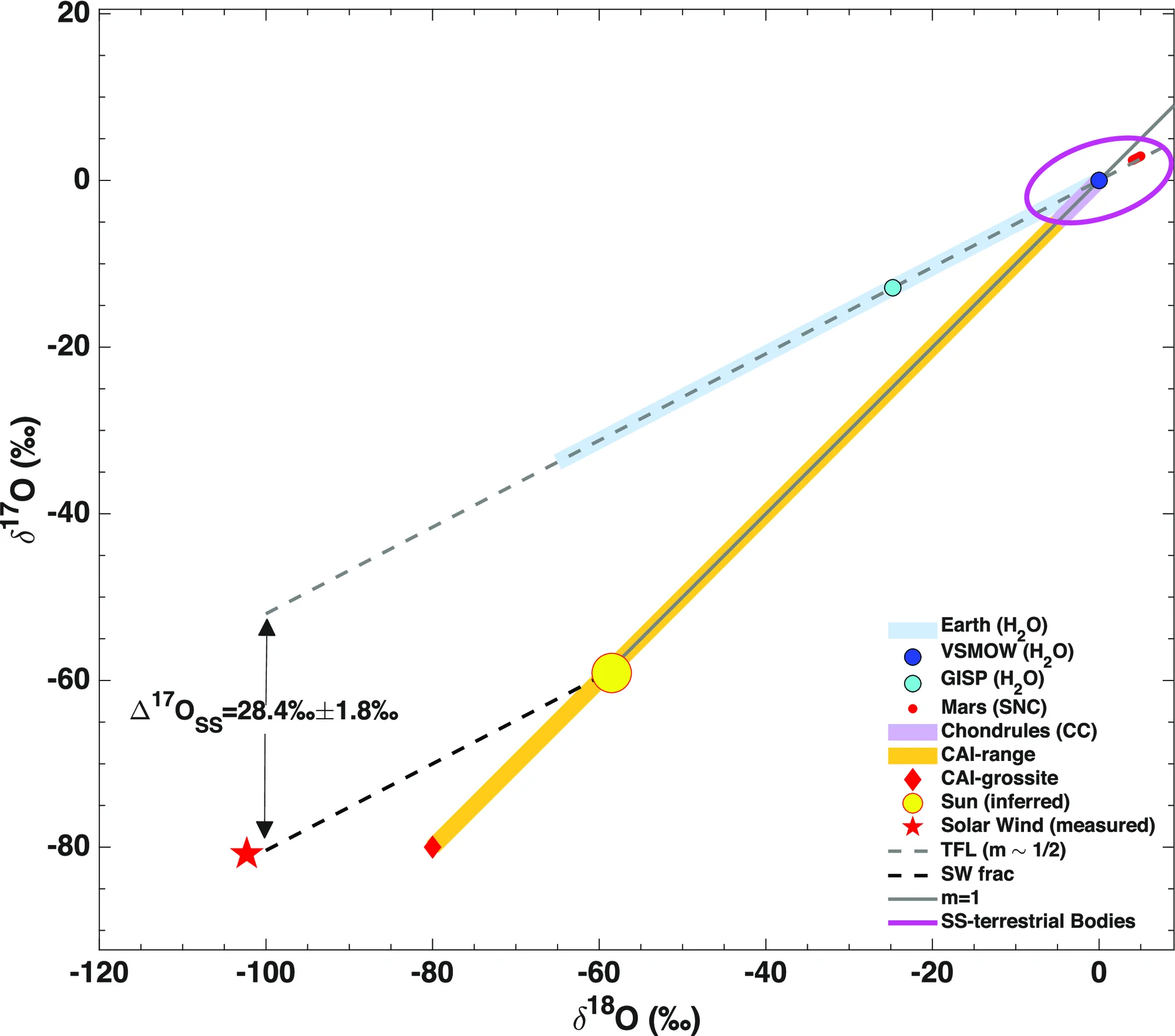
First-order distribution of oxygen isotopes, including major terrestrial H_2_O standards, such as Vienna Standard Mean Ocean Water (VSMOW) and Greenland Ice Sheet Standard Precipitation (GISP) in delta notation. These terrestrial water standards and nonatmospheric oxygen-bearing terrestrial materials help define the Terrestrial Fractionation Line (TFL) for water and is produced by mass-dependent fractionation (MDF) processes. The bulk composition of Mars as inferred from laboratory measurements of SNC meteorites from Mars is slightly depleted in ^16^O compared to Earth’s bulk composition (9). The Sun’s composition as inferred by data from NASA Genesis (2). Ranges of Allende chondrule and CAI compositions also shown as described by ref. 10 and references therein. CAI with grossite inclusions as reported by (11). A key signature associated with MDF is that shifts in the relative abundances of ^17^O/^16^O and ^18^O/^16^O, when quantified with respect to a standard (i.e., VSMOW) using delta notation as δ^17^O and δ^18^O respectively^*^, a plot of δ^17^O vs. δ^18^O defines a line where δ^17^O ~ ½ δ^18^O. This contrasts with the “slope 1” line (m = 1) that connects the bulk composition of the solar system, the Sun, with the terrestrial planets. Processes that selectively inject (or remove) ^16^O, produce slope 1 fractionation patterns that are traditionally quantified as ^17^O anomalies, Δ^17^O = δ^17^O − 0.52 × δ^18^O. In this notation, the Sun’s isotopic composition, normalized to the Earth’s Ocean water (VSMOW), was inferred by the Genesis mission to be approximately given by δ^18^O ~ −58.5‰, δ^17^O ~ −59.1 ‰ and Δ^17^O = −28.4‰ ± 1.8‰. Conversely, when normalized to the Sun’s composition, Earth’s bulk composition (and those of other major solid bodies) are depleted in ^16^O and characterized by oxygen isotopic compositions (Δ^17^O) ranging from ~25 to 32 ‰.

The discovery by NASA’s Genesis mission that terrestrial solids (e.g., planets, asteroids, comets, and the bulk composition of meteorites) are depleted in ^16^O by ~5 to 6% relative to the Sun was unexpected for two major reasons (2). First, interstellar silicate (SiO_2_) dust grains originate from a variety of stellar outflows, each with distinct oxygen isotopic compositions. However, while residing in the interstellar medium (ISM), the isotopic signatures of individual dust grains acquired from their stellar sources are expected to be erased by destructive processes such as galactic cosmic-ray irradiation and supernova shock-wave sputtering and recondensation before they are incorporated into planetary materials (3). Moreover, the isotopic compositions of other major rock-forming elements (e.g., Mg, Fe, and Si) in planetary materials are remarkably homogeneous, typically varying by <5‰ (4–6). Together, these observations strongly support the idea that interstellar dust grains were largely well mixed prior to incorporation into larger solid bodies in the solar system (7). Second, when the ratios of ^17^O/^16^O and ^18^O/^16^O from major solar system reservoirs, including the Sun, are plotted on a three-isotope diagram (Fig. 1), they define an approximately slope-1 relationship that cannot be explained by conventional mass-dependent processes (8). Understanding when, where, and how mass-independent oxygen isotope signatures were generated and incorporated into the building blocks of planets remains a major unsolved problem in planetary formation.

Existing models for generating mass-independent oxygen isotope fractionation generally invoke gas-phase processes, including self-shielded photodissociation and symmetry-dependent chemical reactions involving O, O_2_, and O_3_ (10, 12–15). A defining feature of such processes is the production of slope-1 correlations in three-isotope space, reflecting isotope effects that do not scale with mass differences alone. Fig. 1 illustrates this behavior for solar system materials, which define a mass-independent distribution distinct from the MDF line. Similar slope-1 relationships are also observed in terrestrial atmospheric oxygen reservoirs, where they arise from well-characterized photochemical reactions (8). Whether similar slope-1 relationships can be generated in condensed-phase environments remains an open question.

Here we report laboratory evidence that energetic particle irradiation of icy oxide surfaces produces mass-independent oxygen isotope fractionation in solids at cryogenic temperatures. Control experiments demonstrate that irradiation of solids alone is insufficient to produce the observed effect, indicating that mass-independent fractionation arises from the interaction of radiation-induced defects with water-derived oxygen-bearing species at the solid–ice interface. These results identify a previously unrecognized mechanism for generating MIF in condensed phases and suggest that irradiation-driven processes may influence the oxygen isotopic composition of dust grains in the ISM and the earliest stages of solar system formation.

## Results

### Simultaneous Irradiation.

**Fig. 2.**
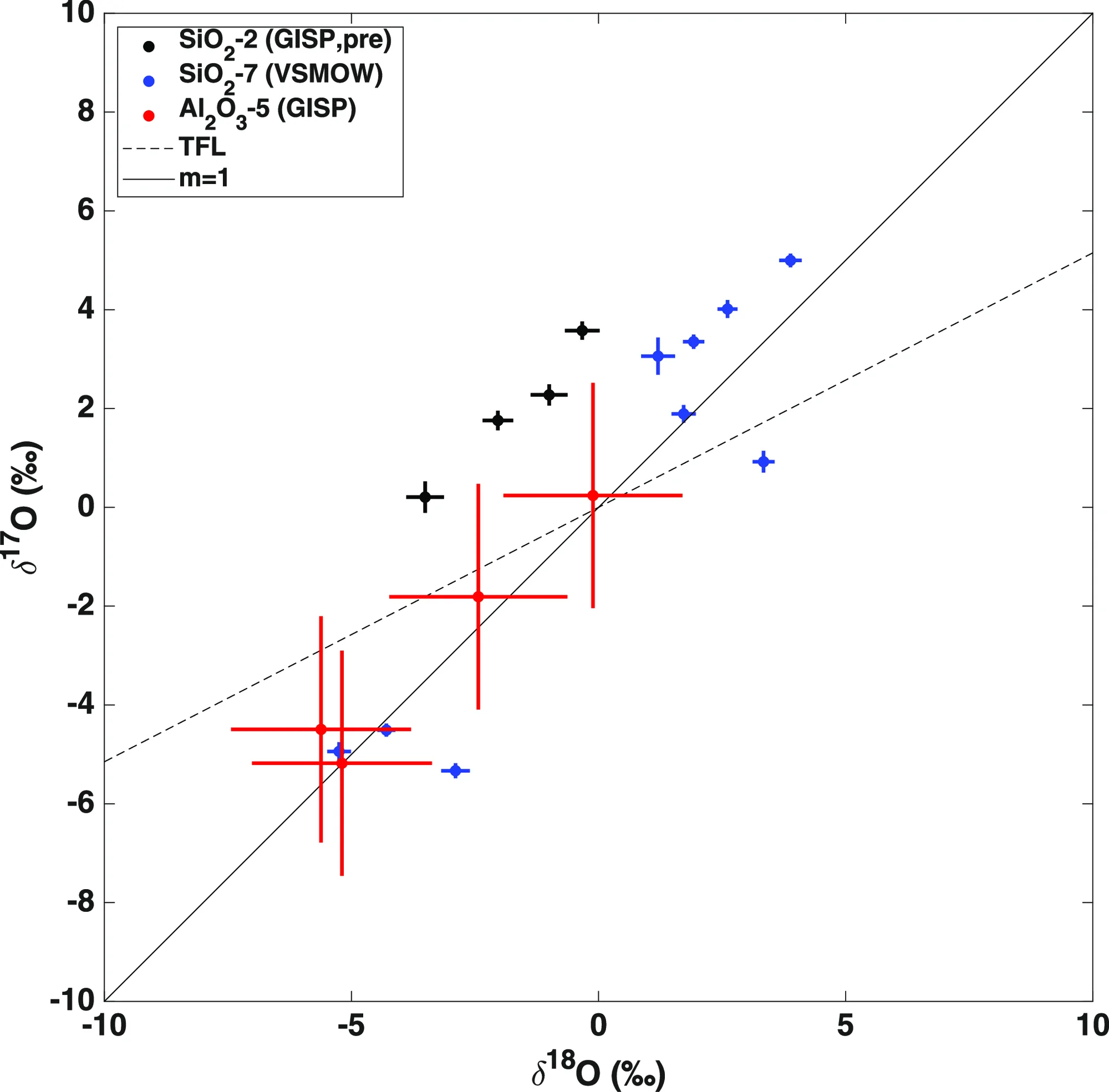
Oxygen three-isotope compositions (δ^17^O vs. δ^18^O) of irradiated SiO_2_ and Al_2_O_3_ surfaces measured by SIMS, reported relative to corresponding unexposed materials. Each point represents an individual analysis spot with 2σ uncertainties. Reference lines corresponding to the TFL (slope ≈ 0.515) and a slope-1 relationship are shown for comparison. The data define a slope-1-like trend within uncertainty (best-fit slope = 0.98 ± 0.27, 2σ), consistent with mass-independent oxygen isotope fractionation produced under low-fluence irradiation conditions. Analytical uncertainties in Δ^17^O are estimated to be ± 0.65‰ for Al_2_O_3_ and ± 0.35‰ for SiO_2_ (see *SI Appendix* for details). Sample numbers (e.g., SiO_2_-2, Al_2_O_3_-5) are laboratory identifiers used to distinguish independently prepared specimens and have no physical significance. Error bars represent 2σ analytical uncertainties determined independently for each sample. Their differing magnitudes reflect variations in counting statistics and SIMS analytical precision among measurements rather than differences in the irradiation experiments. The systematic shift of the SiO2-2 (GISP) is likely the result of the GISP water standard having a more negative δ^18^O (=−24.78 ± 0.09‰) compared to the VSMOW (=0‰) water standard.

Secondary Ion Mass Spectrometry (SIMS) measurements of irradiated H_2_O (ice) covered SiO₂ and Al₂O_3_ surfaces reveal clear and reproducible mass-independent shifts in oxygen isotopic composition relative to unexposed materials. In low-fluence experiments (Fig. 2), SiO₂ and Al_2_O_3_ surfaces exhibit three-isotope relationships consistent with a mass-independent, slope-1-like trend within uncertainty.

**Fig. 3.**
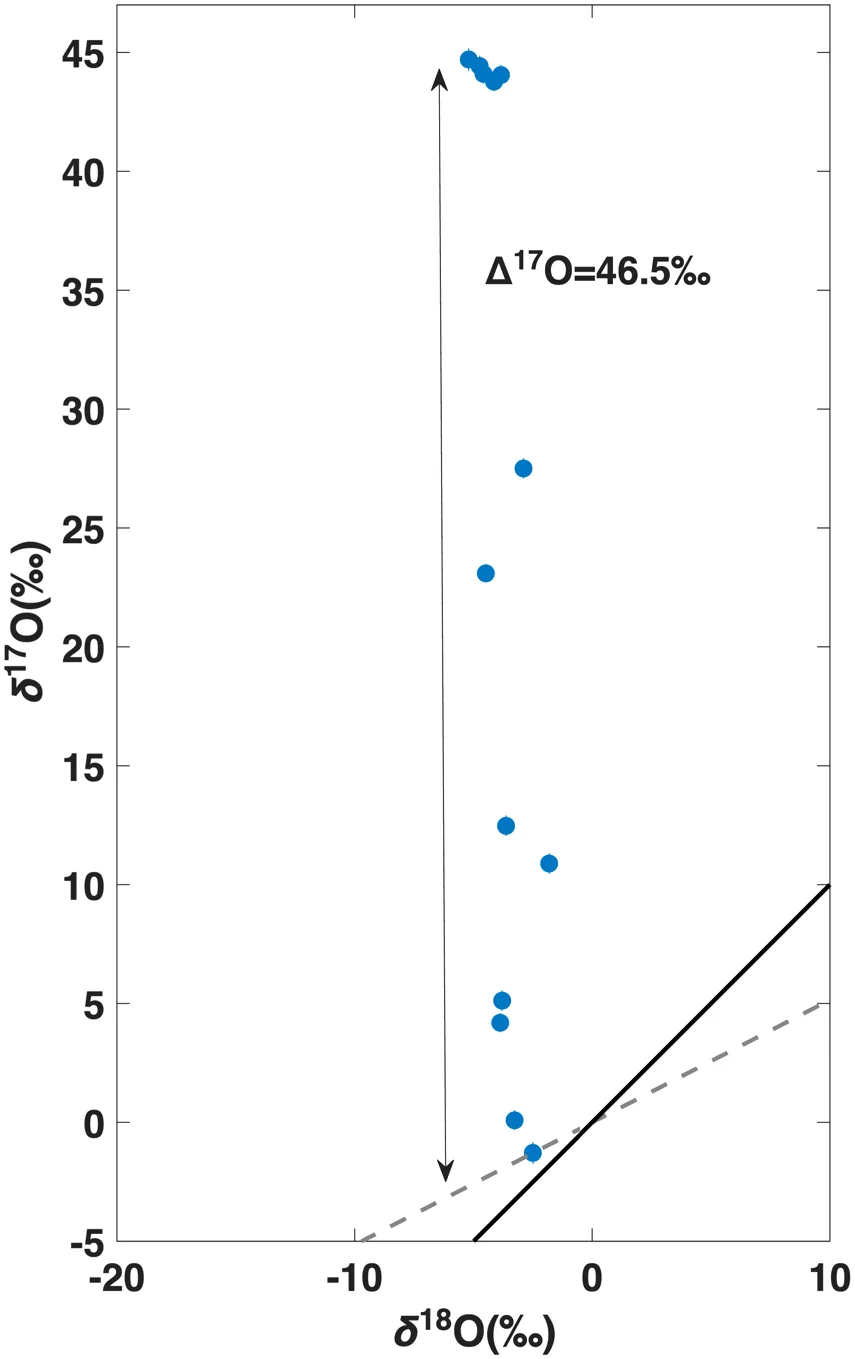
Oxygen three-isotope compositions of an Al_2_O_3_ surface exposed to high electron fluence (~450 min at ~50 μA, T = 10 K). The data show large mass-independent oxygen isotope anomalies (Δ^17^O), with values up to ~46.5‰ for the main cluster and higher for isolated measurements. In contrast to the slope-1-like behavior observed at low fluence (Fig. 2), the high-fluence data follow a distinct trend in three-isotope space. Measurements are taken across multiple analysis locations on the same substrate. These results are consistent with increased defect production and evolving interfacial chemistry at higher energy deposition.

In contrast, H_2_O (ice) covered Al_2_O_3_ surfaces exposed to higher irradiation fluence display substantially larger isotopic anomalies and follow a distinct trajectory in three-isotope space (Fig. 3). The measurements are reproducible across multiple analysis spots and indicate that this behavior is consistent with a substrate-dependent response rather than analytical variability.

### Preirradiation Experiments.

To further constrain the mechanism, we performed experiments in which the oxide substrates were irradiated prior to the deposition of H_2_O ice (pre-). In these preirradiated samples, subsequent exposure to water ice still produces measurable mass-independent oxygen isotope fractionation (Fig. 2). The magnitude and character of the isotope shifts are comparable to those observed in experiments where irradiation and ice are present simultaneously. These results demonstrate that irradiation-induced defects in the solid are sufficient to enable subsequent isotope exchange following exposure to H_2_O ice.

Molecular Hydrogen (H_2_) production was observed after the deposition of H_2_O onto a preirradiated surface (*SI Appendix*, Fig. S6).

### Control Experiments.

For completeness, we carried out several experimental controls to evaluate whether we would have seen isotopic shifts in the solid substrates without the added water and/or e- irradiation. These are summarized in Table 1 below.

**Table 1.** Summary of control experiments and SIMS results

Control # (sample)	Temp.	e^−^ irradiation	H_2_O ice deposition	Δ^17^O (‰)	m	R^2^	Comment
1 (Qz-13)	10 K	0 min	10 mL GISP	−0.84 to 1.61‰	0.81	0.67	Water alone does not produce clear slope 1 effect
2 (Qz-14)	300 K	Pre, 45 min	10 mL GISP, after e^−^	−0.49 to 0.88‰	0.46	0.84	Room temperature irradiation does not enhance effect or produce slope 1 effect

### Defect Formation Timescales.

We estimated defect formation timescales in the early solar system and in the ISM (*SI Appendix*, section S3). These calculations show that a substantial fraction of oxygen-bearing bonds in dust grains are expected to be broken in these astrophysical environments (Fig. 6).

## Discussion

The experiments demonstrate that energetic particle irradiation of icy oxide surfaces produces mass-independent oxygen isotope fractionation in solids under cryogenic conditions. Because thermal energy at 10 K is insufficient to break Si–O or Al–O bonds (16), the observed isotope effects require irradiation-induced bond breaking followed by isotope-selective reactions involving oxygen-bearing species. In low-fluence experiments, both SiO_2_ and Al_2_O_3_ exhibit three-isotope relationships consistent with slope-1-like behavior, indicating that irradiation-driven processes can generate anomalous oxygen isotope effects across different oxide substrates. At higher fluence, Al_2_O_3_ develops substantially larger isotopic anomalies (Fig. 3), consistent with increased energy deposition and defect production. Together, these observations establish energetic-particle irradiation as a previously unrecognized pathway for generating mass-independent oxygen isotope signatures in condensed phases.

A key feature of our results is the dependence of isotope systematics on both irradiation conditions and substrate composition. While both SiO_2_ and Al_2_O_3_ exhibit slope-1-like behavior at low fluence, Al_2_O_3_ displays larger anomalies and distinct trends in three-isotope space at higher fluence (m ~ −5). This behavior suggests that the initial generation of anomalous fractionation may be broadly similar across substrates, but its subsequent evolution depends on substrate-specific defect formation and interfacial reaction pathways. These findings support a framework in which MIF arises from defect-mediated interfacial chemistry rather than from a single universal reaction pathway. Additional studies are needed to clarify the mechanism(s) involved at higher fluences.

Several reaction pathways may contribute to the observed isotope effects, including the formation and subsequent reactions of species such as O, O_2_, O_3_, OH, and H_2_O_2_ in irradiated ice (8, 17–20). In analogy with gas-phase MIF reactions (15), symmetry-dependent isotope effects involving transient excited intermediates (e.g., O_3_*) may also contribute to the condensed-phase chemistry we report here; however, the presence of a solid interface introduces additional constraints and opportunities for isotope-selective reactions. The relative importance of these pathways likely depends on both substrate properties, temperature, and irradiation conditions.

Although the present experiments clearly demonstrate that irradiation-driven defect chemistry can generate mass-independent oxygen isotope fractionation in condensed phases, they do not yet uniquely identify the microscopic reaction pathway responsible for the observed isotope effects. Future experiments designed to isolate individual oxygen-bearing reactants and reaction intermediates will help distinguish among competing mechanisms involving defect-mediated exchange, symmetry-dependent chemistry, and other excited-state processes.

An additional constraint on the mechanism is provided by experiments in which irradiation precedes the introduction of water ice. In these “preirradiated” samples, mass-independent oxygen isotope fractionation is still observed following subsequent exposure to H_2_O, indicating that defect formation in the solid is sufficient to enable anomalous isotope chemistry. The observation of H_2_ formation following H_2_O deposition onto preirradiated surfaces indicates that radiation-induced defects store chemical energy capable of driving bond-breaking reactions well after irradiation has ceased and demonstrates that the generation of mass-independent fractionation does not require simultaneous irradiation and ice chemistry but instead can arise from reactions between preexisting radiation-induced defect sites and later-arriving oxygen-bearing volatile species. Future experiments with other volatile O molecules, such as O_2_, will help to further constrain the MIF mechanisms reported here.

This behavior further supports a framework in which defect-mediated interfacial chemistry plays a central role in controlling isotope fractionation. In this picture, irradiation establishes a population of reactive sites whose subsequent interaction with volatile species governs the magnitude and character of the isotope effect. This decoupling of defect formation and MIF isotope chemistry suggests that irradiation may prime solid surfaces for later isotopic modification in environments where ice deposition and radiation exposure occur on different timescales (e.g. in molecular clouds and the diffuse ISM).

**Fig. 4.**
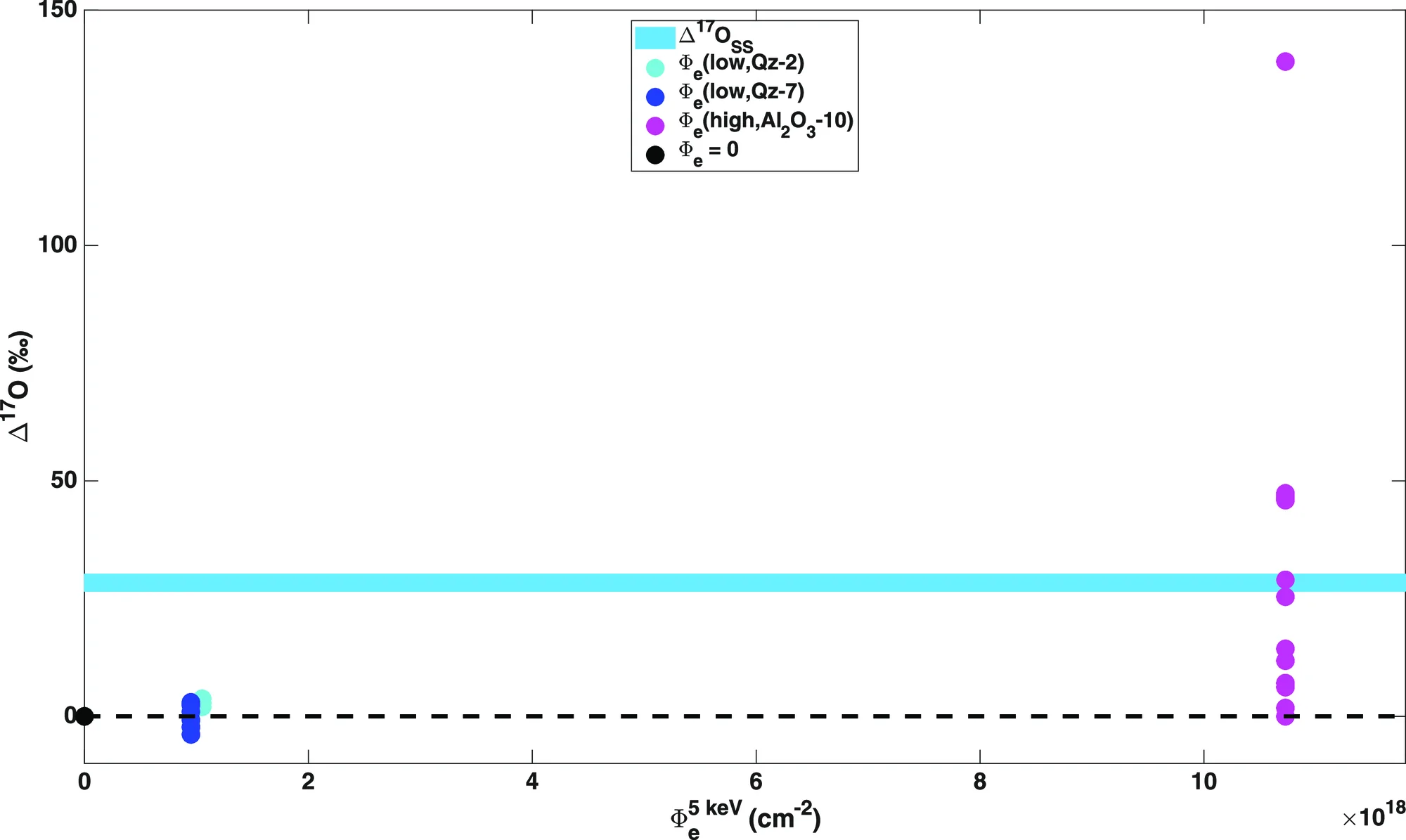
Relationship between mass-independent oxygen isotope anomaly (Δ^17^O) and electron fluence for irradiated oxide surfaces. Increasing irradiation fluence produces progressively larger isotope anomalies, consistent with increased energy deposition and defect production within the solid. These results indicate that the magnitude of mass-independent fractionation is controlled, at least in part, by cumulative radiation dose. The shaded band (Δ^17^O_ss_) denotes the approximate Δ^17^O difference separating the Sun from bulk planetary materials as measured by Genesis (1).

**Fig. 5.**
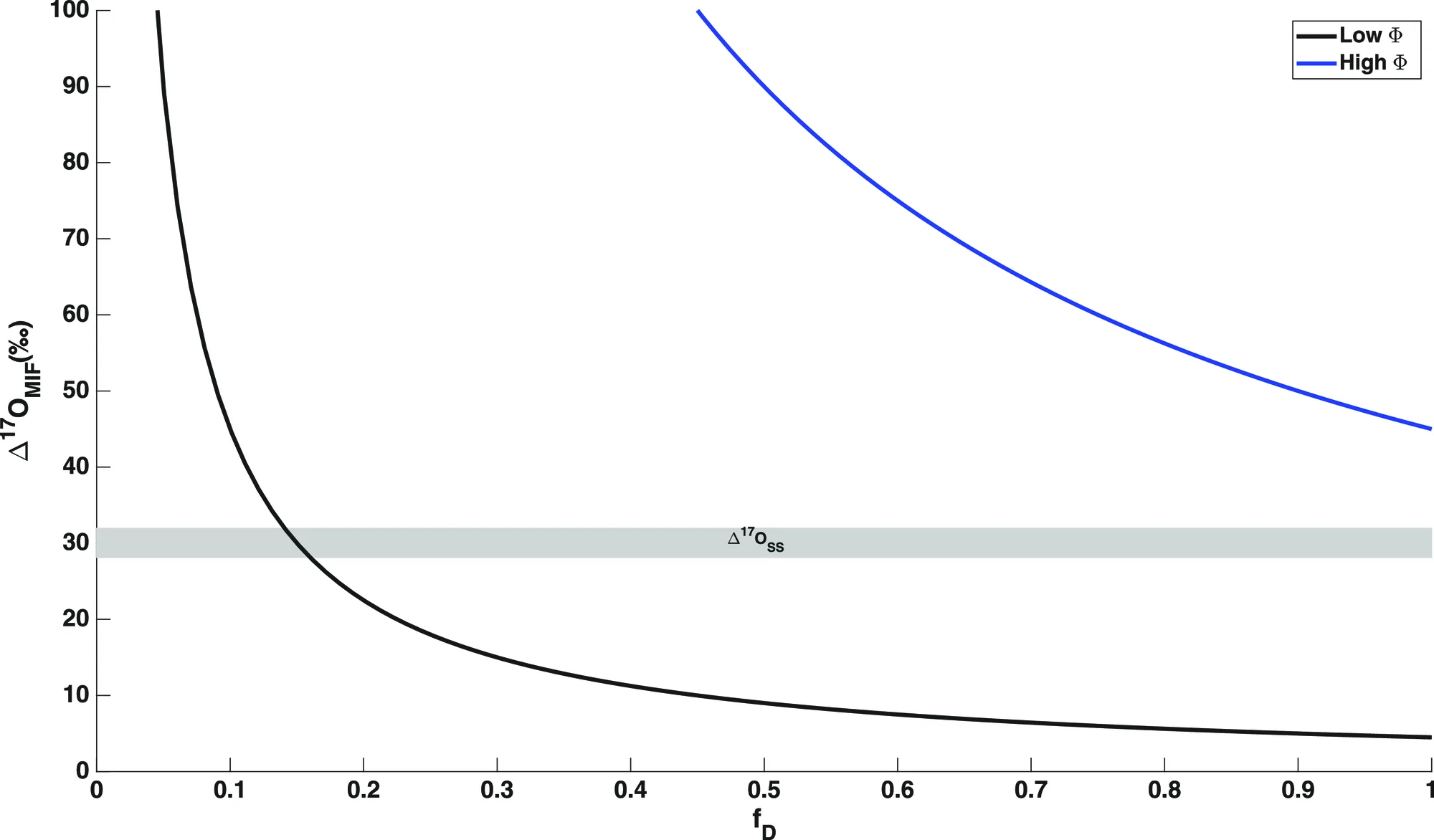
Estimated instantaneous mass-independent fractionation factor (Δ^17^O_MIF_) as a function of the fraction of metal–oxygen bonds (f_D_) participating in isotope exchange. Values are inferred from SIMS measurements of irradiated surfaces using a mass-balance framework (*SI Appendix*). The shaded region indicates the range of Δ^17^O values that separate the Sun from terrestrial planetary materials (1). The results demonstrate that the instantaneous fractionation factor of radiation induced mass-independent fractionation is significant.

The experimental results indicate that energy deposition in the solid produces radiation-induced defects that facilitate oxygen isotope exchange between the oxide and surrounding oxygen-bearing species. Molecular dynamics simulations suggest that only a fraction of bonds within the irradiated volume are disrupted at low fluence (*SI Appendix*, Fig. S1), implying that the observed isotope anomalies arise from partial exchange between altered regions of the solid and the volatile oxygen-bearing ice. Consistent with this interpretation, the magnitude of Δ^17^O increases with electron fluence (Fig. 4), indicating that the extent of isotope fractionation is controlled, at least in part, by cumulative energy deposition and defect production. In addition, the relationship between Δ^17^O and the extent of defect-mediated exchange (Fig. 5) suggests that the instantaneous fractionation factor of the anomaly is larger than the measured isotopic shifts (Fig. 5). Together, these results highlight the efficiency of defect-mediated processes in modifying the isotopic composition of solids under cryogenic conditions.

Laboratory irradiation experiments necessarily employ particle fluxes higher than those found in most natural environments to reproduce long-term energy deposition within practical timescales. In this context, the relevant parameter governing radiation-driven chemistry is the total deposited energy rather than instantaneous flux. While the electron currents used here exceed typical cosmic-ray fluxes, energetic ion deceleration in solids is expected to generate cascades of secondary electrons with energies sufficient to disrupt chemical bonds and produce defects (1). Accordingly, these experiments are designed to identify irradiation-driven chemical pathways rather than replicate specific environmental conditions. A similar argument can be applied to the vacuum pressures of the experiments we report here. The control experiments show that the anomalous oxygen isotopic results are the result of having both electron irradiation and H_2_O ice present in the experiments (*SI Appendix*, section S6).

**Fig. 6.**
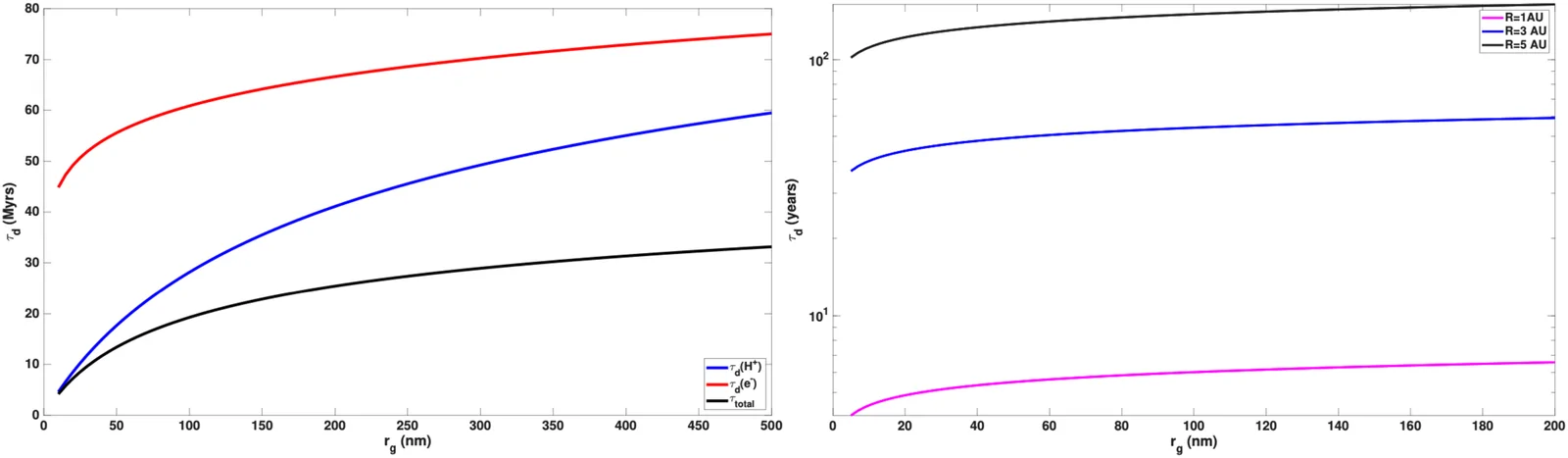
Defect formation timescale (1/e) as a function of dust grain radius. Estimated timescales for defect formation and subsequent oxygen isotope exchange in astrophysical radiation-processed environments. (*Left*) Timescales for defect formation on dust grains in the ISM are on the order of tens of millions of years (*SI Appendix*, section S3). Defect formation during the T-Tauri phase, where enhancements in solar cosmic rays are expected, are much shorter and a function of distance from the Sun. These estimates support the plausibility of irradiation-driven isotope modification under natural conditions. For reference, the residence time of interstellar dust grains in the ISM are ~400 My (21).

The ability of irradiation to generate large mass-independent oxygen isotope anomalies in solids has important implications for environments in which icy materials are exposed to energetic particles, including interstellar dust grains and early solar system solids. In such settings, defect-mediated surface chemistry may provide a pathway for coupling the isotopic composition of solids and volatiles. Estimates of defect-formation timescales (Fig. 6) further suggest that once radiation-induced defects are established, isotopic modification of solids may occur on timescales (~10^1^ to 10^2^ y in PPDs and <10^2^ My in the ISM) that are short relative to the residence time of dust grains (~1 My in PPDs and 10^2^ to 10^3^ My in the ISM) in radiation-exposed environments. While additional work is required to quantify the efficiency and extent of this process in natural systems, the results presented here demonstrate that irradiation-driven processes provide a viable mechanism for generating mass-independent oxygen isotope heterogeneity under cryogenic conditions.

## Materials and Methods

Electron irradiation induced surface chemistry experiments at T = 10 K were carried out at CSU San Marcos. Electron irradiation times of ~45 min (low e- fluence) and ~450 min (high e- fluence) were done using an electron gun (Kimball Physics) beam current of ~50 μA and energies of 5 keV/electron. We conducted irradiation experiments with relatively low and high fluences (~45 and ~450 min.) using a variety of targets and water compositions. Changes in the oxygen isotopic compositions of the solid surfaces were evaluated and quantified by using the SIMS at University of California–Angeles. We focused on quantifying shifts in the isotopic composition of irradiated solid surfaces since shifts in the isotopic composition of the water-ice reservoir (N_H2O_ > 3 × 10^18^) were expected to be small compared to those of metal oxide solid targets (N_M-O_ ~ 2 × 10^13^) affected by the irradiation. Additional details, including instrumental blanks, and controls are found in *SI Appendix*.

## Supplementary Material

Appendix 01 (PDF)

## Data Availability

The data and Matlab code used to process the data have been deposited in Zenodo (22). All other data are included in the article and/or *SI Appendix*.

## Footnotes

^*^A note on δ^x^O notation: It is typical in isotope geochemistry to denote the abundance of an isotope of an element using “delta” notation. For any element X with major isotope of mass i (^i^X) and minor isotope of mass j (^j^X), comparison of the relative ratio of these two isotopes compared to a well-known standard is quantified as a deviation from this standard as:

$$\delta^{j}X\;=\;[(\mathrm{Rsample}(i,j)/\mathrm{Rstandard}(i,j)\;-\;1]\;\times \;1,000,$$

where the ratio of abundances of isotopes is given by R_y_(i,j) = [^j^X]/[^i^X] (y = sample, standard). In the case of oxygen, the anomalous or mass-independent ^17^O content of an object is quantified as the deviation from the expected mass-dependent behavior as:

$$\Delta^{17}O\;=\;\delta^{17}O(\mathrm{meas}.)\;-\;0.52\;\times \;\delta^{18}O(\mathrm{meas}.).$$
