# Supplementary material for: Mass-independent oxygen isotope fractionation driven by energetic-particle irradiation of icy solids
Source: Proc Natl Acad Sci U S A. 2026 Oct 21;123(43):e2610806123. doi: 10.1073/pnas.2610806123 (PMC13715750; doi:10.1073/pnas.2610806123)
Supplement: Supplementary file 1 [file pnas.2610806123.sapp.pdf]

Supporting Information for

Mass-Independent Oxygen Isotope Fractionation Driven by Energetic Particle Irradiation of Icy Solids

Gerardo. Dominguez, Caroline Caplan, Lauren Tafla, Joshua Lucas, Ming-Chang Liu, Kevin McKeegan, Jeff Gillis

Corresponding Author: Gerardo Dominguez
Email: gdominguez@csusm.edu

This PDF file includes:

- Supporting text
- Figures S1 to S6
- Tables S1 to S3
- SI References

Supporting Information Text

S1. Mass-balance model for isotope exchange

We describe a simple mass-balance framework to interpret the oxygen isotope composition of irradiated solids in terms of defect-mediated exchange.

Molecular Dynamics (MD) simulations of radiation damage applied to our experiments suggest that only a small fraction (f_D) of the Si-O bonds in the irradiated volume were broken by the electron beam in the low-fluence experiments (See Section S2). These defects would be susceptible to being re-oxidized by any oxygen-bearing volatile compounds found on the surface. Using mass balance and the assumption that there was a ^{16}O depleted and, by mass-balance, a ^{16}O enriched reservoir of equal magnitude ($+\Delta^{17}O_{MIF}$, $-\Delta^{17}O_{MIF}$) on the surface, we write the oxygen isotopic composition of a SIMS analytical volume as:

$$<\Delta^{17}O_{SIMS}>_V = f_+ \cdot \Delta^{17}O_{MIF} + f_- \cdot (-\Delta^{17}O_{MIF}) + (1 - f_D) \cdot \Delta^{17}O_{blank} \quad (1)$$

where f_+ ($=f_D - f_-$) and f_- are the fraction of broken Si-O bonds (f_D) in the SIMS analytical volume that were re-oxidized by the $+\Delta^{17}O_{MIF}$ and $-\Delta^{17}O_{MIF}$ reservoirs respectively and $\Delta^{17}O_{blank}$ is the anomaly of the unaltered sample ($\Delta^{17}O_{blank} = 0$). We used this interpretation to obtain a *lower limit* on the instantaneous fractionation factor ($D^{17}O_{MIF}$) associated with the ^{16}O depleted reservoir that oxidized broken Si-O bonds in the low fluence experiments. Setting $f_-=0$ and solving equation (1) for $+D^{17}O_{MIF}$ to get:

$$\Delta^{17}O_{MIF} \geq \frac{\max(<\Delta^{17}O_{MIF}>_V)}{f_D} \quad (2)$$

$$\Delta^{17}O_{MIF} \geq \frac{(3.5 \pm 1)}{f_D} ‰ \quad (3)$$

We note that the equality corresponds to the case where $f_D=1$. Estimates of f_D using Molecular Dynamics simulations of electron energy deposition and defect production in quartz targets (See Section S2) suggests that f_D may be as low as 0.10 in the low fluence experiments, which implies that $\Delta^{17}O_{MIF} > 35$ ‰. Additional experiments are needed to better constrain f_D.

S2. Molecular dynamics simulations

Molecular dynamics simulations were used to estimate the fraction of bonds disrupted during irradiation and to constrain the extent of defect formation under experimental conditions.

Radiation Defects in Quartz. The creation of defects in solids like quartz has been studied experimentally and theoretically. While there remain uncertainties in this understanding, it appears that the net effect of energy losses by ions in solids is to permanently disrupt the structure of ordered materials like quartz, leading to the creation of several kinds of “defects”. Wang et al. (1), using Molecular Dynamics (MD) to simulate radiation damage caused by ballistic energy inputs into α–quartz, note that in addition to oxygen vacancies, which are associated with the displacement of oxygen atoms from their lattice positions, radiation damage also produces over-coordinated oxygen and five-coordinated Si atoms(1).(1). While it is unclear how the various types of defects contribute to the isotopic signals we observe in our low and high fluence

experiments, here we focus on evaluating the plausibility of creating atomic dislocations of the type that would be most susceptible to interactions with volatile oxygen reservoirs on the surface.

First, using conservation of energy and momentum, we estimate the maximum energy of a head-on collision between an electron (m_e) and a ^{16}O atom ($16m_p$), in what is referred to as a Primary Knock-on Atom (PKA) event. This maximum recoil energy, for an electron with E=5 keV colliding head on with a ^{16}O atom, is given by:

$$E_R = \frac{1}{2}\left(16m_p\right) v_R^{\ 2} \tag{4}$$

where the recoil velocity (v_R) is given by:

$$v_R = \frac{(2m_e)}{(m_e + 16m_p)}\left(\frac{2E_e}{m_e}\right)^{1/2} \tag{5}$$

Combining equations 4 and 5 we get the maximum recoil energy of an oxygen PKA, which is given numerically by:

$$E_R = 0.68\ \left(\frac{E_e}{5keV}\right)^{1/2} eV \tag{6}$$

For a 5 keV electron, this maximum recoil energy is approximately given by 0.68 eV.

Using the semi-empirical power-law scaling of defect creation previously reported (1), given by:

$$N_d = AE_R^{\ m} \tag{7}$$

we calculated the maximum number of (predicted) PKA-defects and plot these in Figure S1 below. The maximum value for E_R assumed that a 5 keV electron collides head-on with a target nucleus and the parameters A= [0.539 1.225 2.104 2.820] and m= [0.72 0.80 0.89 0.88] correspond to Si^{III} (oxygen vacancy), O^{I}, Si^{V}, and O^{III} type defects.

To calculate the fraction of total target volume affected by defects (f_D), we assumed that the thickness of the 5 keV electron beam damage was ~250 nanometers, consistent with CASINO calculations (See Figure S1 and next section) of electron energy loss in SiO_2.

There appears to be semi-quantitative correspondence between the fraction $\mathbf{f_D}$ of the solid that we deduce has been altered by the electron irradiation and exposure to H_2O and the total number of defects predicted by MD simulations of radiation damage in quartz. Reconciling these two numbers is beyond the scope of this paper but could be a fruitful avenue of research as MD simulations and models would benefit from the empirical calibrations made possible by our experimental approach.

CASINO Simulations of electron beam damage. We estimated the regions of e-beam damage using a Monte-**CA**rlo **SI**mulations of electro**N** trajectory in s**O**lids (CASINO) software v.3.3.0.4 (2). Figure S2 shows that 5 keV electrons are expected to cause electron beam damage down to a depth of 200-250 nm in SiO_2.

S3. Radiation energy deposition and astrophysical relevance

We assess the relevance of laboratory electron irradiation to astrophysical environments by comparing energy deposition processes in solids.

Relevance of 5 keV electrons. While the energy of electrons used in our experiments (5 keV) are not as high as the energies of galactic or solar cosmic rays (E>10 MeV), we believe that our experiments adequately simulate the essential features of cosmic-ray induced damage in solids. First, relativistic protons moving through solids lose energy primarily via Coulomb interactions with electrons bound to atoms in the lattice(3). For a typical cosmic ray and interstellar dust grains (r_g~200 nm), this energy loss is consistent with the energy of electrons we used (~10^2-10^3 eV). Cosmic ray particle (e^-, H^+, He^{2+}) interactions with solids produce a cascade of secondary electrons with lower but sufficient energies (~10-10^4 eV**)** to disrupt chemical bonds, producing defects. This cascade of secondary electron production is expected to continue until an energetic threshold (E^*) is reached and the secondary electrons no longer have the energy required to dislocate atoms or other electrons. Thus, while our experimental work makes use of electrons, we believe that future work with protons as the source of secondary electrons are likely to yield similar results to the ones we report here.

Cosmic ray Fluxes and Timescale for Defect Formation. We estimated the timescales for oxygen isotope exchange in the interstellar medium (ISM) and in the early solar system (ESS) by focusing on the production of defects contributed by the proton (H^+) components of cosmic rays (CRs) in each of these environments. For the ISM, we used the cosmic-ray energy spectrum ($S(E_{CR})$) derived from the analysis of the Voyager spacecraft (4) for energies ranging from 1 MeV (E_{min}) to 10 GeV (E_{max}). While this energy range is rather large, the power-law nature of this distribution translates in a cosmic ray exposure rate ($\Phi_p = 2.94\times 10^5$ cm^{-2} s^{-1}) in the ISM with an average energy of ~40 MeV.
To estimate the rate of defect production, we first determined how much energy a single cosmic ray with energy E_{CR} loses while traversing a dust grain of radius r_g. Using empirical determinations of energy-loss ($\frac{dE(E_{CR})}{dz}$) of protons in SiO_2, we estimated the energy deposited in a dust grain of radius r_g as:

$$E_D(E_{CR}, r_g) \simeq 2r_g \frac{dE(E_{CR})}{dz} \qquad (0.8)$$

For example, the energy loss per unit length of a 40 MeV proton in SiO_2 is approximately 39.23 MeV/cm which translates into a total energy loss (deposited) of ~790 eV into a 200 nm in radius dust grain. ***It's important to note that cosmic rays are able to pass-through typical interstellar dust grains without losing a significant amount of their energy (790 eV out of 40 MeV in this case) and the radiation damage caused by cosmic rays is due to electrons with similar energy to those used in our experiments.***

To estimate the rate of defect formation ($\frac{dN_D}{dt}$), we took two approaches. In the simplest approach, we assume that the production of a single atomic defect takes a threshold energy (or work function) of (E_{th}) and the number of defects produced is given by

$$N_D \sim \frac{E_D}{E_{th}} \qquad (0.9)$$

Thus, a single 40 MeV CR proton may be expected to produce ~8 defects in the entire volume of a 200 nm dust grain if E_{th}=100 eV. In the second approach, we used the scaling laws of the MD simulations to determine the defect formation rate as a function of the energy of deposition E_D. For comparison, this semi-empirical model predicts a total number of ~21 atoms, with ~12 of these being oxygen atoms**. In general, the simple-minded and model approaches produced defect formation rates that were within a factor of 2-3 over the entire CR energy spectrum.**

The overall rate of defect formation was calculated by integrating over the cosmic-ray energy spectrum. This flux weighted defect production rate for a dust grain of radius r_g is given by

$$\frac{dN_D}{dt} = 4\pi \int_{E_{\min}}^{E_{\max}} N_D(E, r_g) \frac{dJ(E_{CR})}{dE} r_g^2 dE_{CR} \qquad (0.10)$$

Finally, the oxygen isotope exchange timescale due to cosmic-ray irradiation is given by:

$$\tau_D(r_g) = \frac{N_O(r_g)}{\frac{dN_D(r_g)}{dt}} \qquad (0.11)$$

Where N_O is the number of oxygen atoms found within the dust grain. For simplicity, we assumed that the number of oxygen atoms in a dust grain of radius r_g is given by:

$$N_O(r_g) \sim 2\times \frac{\frac{4\pi}{3}\rho_g r_g^3}{60 m_h} \qquad (0.12)$$

for a pure SiO_2 dust grain composition where each SiO_2 molecular unit has a single Si atom and two ^{16}O atoms.

A summary of the major variables and terms used in these calculations is given in Table S1.

Cosmic-ray energy Spectra Used. To estimate the isotope exchange timescales in the ISM and early solar system, contemporary solar system and early solar system we used the cosmic ray electron spectrum $\frac{dJ}{dE}$ as described in (5) and the corresponding energy loss functions described by (6) This energy spectrum is reproduced in Figure S3.

A separate source of energetic particles with energies > 10 MeV are Solar Energetic Particle events (SEPs) associated with Coronal Mass Ejections. The abundance of short-lived radionuclides in meteorites cannot be explained by contemporary solar CR fluxes ($\Phi_p \sim 150$ protons cm^{-2} s^{-1}). However, similar to T-Tauri stars characterized by their enhanced solar activities, it is likely that the young Sun in the early solar system may have been more active by up to a factor of ~ 10^5. To estimate the solar CR flux experienced by dust grains in the early solar system, we followed an approach similar to (7) and scaled the cycle averaged CR energy spectrum for event 1 in (5) by a factor of ~10^5 to give us a T-Tauri solar cosmic-ray energy spectrum for protons ($S_{TT}(E_{CR})$) and a total flux of protons with energies > 10 MeV of $\Phi_p(E > 10 MeV) = 1.5x10^7 cm^{-2}$ s^{-1} at 1 a

distance of 1 AU from the Sun. The CR spectrum (T-Tauri) used in our calculations of the oxygen isotope exchange timescales is reproduced in **Figure S3** while the timescales as a function of dust grain size and distance from the Sun are plotted in the main text **(Figure 6).**

Estimating Defect and Oxygen Exchange Rates and Timescales. We used the Voyager results of (4) to calculate cosmic ray proton (H^+) and electron (e^-) fluxes and the timescale (τ_D) needed for cosmic rays to break a significant number of bonds in interstellar dust grains. Using data of galactic cosmic ray electrons and proton intensities (J) as a function of energy E presented in (4) we calculated the number of defects produced by CR protons and electrons as follows. The amount of energy deposited per CR ion into a dust grain of diameter $D_g (= 2r_g)$ is given by:

$$\Delta E_{CR} = \left(\frac{dE}{dx} (E_{CR}) \right) D_g \tag{13}$$

where $\frac{dE}{dx}$ is the energy-loss or a cosmic ray ion per unit length traversed in the dust grain material.

Every discrete CR ion interaction is expected to lead to energetic electron production within the dust grain volume. We estimate the number of defects created by these interactions by applying equation 12 to estimate the number of defects as:

$$N_D(\Delta E_{CR}) = A(\Delta E_{CR})^m \tag{14}$$

Therefore, the rate of defect formation in a dust grain of radius r_g caused by CRs of energy E is given by:

$$\left(\frac{dN_D}{dEdt} \right)_{Total} = J(E) \cdot N_D(\Delta E_{CR}) \cdot 4\pi r_g^2 \tag{15}$$

where $J(E)$ is given in units of number of cosmic rays per steradian per MeV per second.

We integrated this expression to obtain the total defect production rate for all cosmic ray energies in a dust grain of radius r_g as follows:

$$\left(\frac{dN_D}{dt} \right)_{Total} = \sum_{E_{CR}} J(E_{bin}) \cdot N_D(\Delta E_{CR}) \cdot 4\pi r_g^2 \cdot \delta E \tag{16}$$

where $\delta E = (E_{\min} - E_{\max})$, $E_{bin} = E_{\min} + (E_{\max} - E_{\min})/2$, and the sum is performed over the range of energies for a given cosmic-ray energy intensity spectrum $J(E)$ as shown in Figure S3.

Finally, the characteristic timescale (1/e) for significant defect formation in a dust grain of radius r_g is given by:

$$\tau_D = \frac{N_{O-atoms}}{\left(\frac{dN_D}{dt} \right)_{Total}} \tag{17}$$

where $N_{O\text{-atoms}}$ is the number of oxygen atoms in an SiO_2 dominated dust grain.

For the ISM, we tabulated the rates of defect formation as well as the corresponding timescales separately for protons and electrons and combined these to obtain the total defect timescale as:

$$\tau_D(total) = \left(\frac{1}{\tau_D(H^+)} + \frac{1}{\tau_D(e^-)} \right)^{-1} \quad (18)$$

The results of these numerical calculations of defect formation timescales for dust grains as a function of their radius (r_g) are given in Figure 5 in the main text.

We performed more detailed estimates of exposure times as follows. Galactic and solar cosmic ray proton and electron energies, on average, exceed 10 MeV. At these energies, the stopping length (>10^{-2} m) of typical CRs greatly exceeds the typical size of interstellar dust grains (r_g~10^{-7} m) (3). Thus, the relevant energy for evaluating how much radiation damage is experienced by dust grains is the energy deposited per cosmic ray ($E_D \sim \frac{dE}{dx} 2r_g$) integrated over the spectral intensity ($\frac{dJ}{dE}$) of cosmic rays. For example, the dE/dx of a 40 MeV proton in SiO_2 is ~39.23 MeV/cm, which translates into an energetic deposition of ~790 eV in a 200 nm dust grain per cosmic ray proton interaction(3).(3). Adopting a simple energy threshold model that requires 100 eV of energy deposition per defect, we estimate that a single CR proton can produce between 8-9 atomic dislocations (N_D) in a dust grain with r_g~200 nm.

A more detailed analysis that considers the spectral intensity of cosmic ray protons together with the CR energy dependence of energy loss and defect formation in SiO_2 is detailed below. In this more careful analysis, the rate of defect formation a dust grain of radius r_g is given by:

$$\frac{dN_D(r_g)}{dt} = 4\pi \int_{E_{\min}}^{E_{\max}} N_D(E_{CR}, r_g) \frac{dJ(E_{CR})}{dE} r_g^2 dE_{CR} \quad (19)$$

where N_D (E_{CR}) is the number of defects produced by a cosmic ray with energy E_{CR} traversing a silicate dust grain with radius r_g. The (1/e) timescale for resetting the Si-O bonds of a water-ice covered SiO_2 dust is given by:

$$\tau_D = \frac{N_O}{\left(\frac{dN_D}{dt}\right)_{Total}} \quad (20)$$

where N_O is the number of oxygen atoms in a dust grain. Our analysis finds that the timescale for producing dust grains susceptible to becoming ^{16}O depleted solids early in the solar system may have rapid (10^1-10^2 years) at the edges of regions in the early solar system where dust grain opacity is negligible and beyond the snowline (R>3AU, See Figure 6). Defect formation timescales in the ISM are ~ 10^5 times slower (1-10 Myrs) but the typical of interstellar silicate grains is on the order of 400 Myrs (8) and the lifetime of molecular clouds is only ~10 Myrs. This suggests that the population of interstellar dust grains that eventually became the solar system's solids may have already been ^{16}O depleted compared to the local ISM's bulk composition prior to the solar system's formation.

S4. Estimation of exchange fraction and isotope anomaly magnitude

The magnitude of the observed isotope anomalies can be related to the fraction of oxygen atoms participating in exchange through a simple mixing framework as laid out in Section S1.

That analysis showed that a lower limit to the magnitude of the instantaneous mass-independent fraction is $\Delta^{17}O_{MIF} > 35‰$.

The high fluence experiment provides additional evidence that the actual magnitude of the ^{16}O depleted carrier is much larger than 3.5‰. Here, we found that an increase in the exposure duration of irradiation by 5 keV electrons by a factor of ~10 increased the amount of ^{16}O depletion recorded in the solid by a similar factor, as evidenced by several SIMS spots with measured isotopic anomalies ($\Delta^{17}O_{SIMS}$) as large as ~139‰. To our knowledge, these spots represent some of the largest oxygen isotopic anomalies transferred to metal-oxides observed in the laboratory and imply that the instantaneous fractionation factor associated with the mass-independent chemistry on the cold surfaces had $\Delta^{17}O_{MIF} \geq 47.3‰$ We plot $\Delta^{17}O_{MIF}$ as a function of f_D and compare these values to the ^{16}O depletion that characterizes the bulk of the solar system's terrestrial solids ($\Delta^{17}O_{SS}$) as reported by the Genesis mission in Fig.4. These results suggest that physical-chemical process(es) can occur at temperatures as low as 10 K and may contribute to dust grains with the systematic ^{16}O depletion observed in the solar system. We summarize the oxygen isotopic anomalies observed in our experiments as a function of electron fluence in Figure 5.

S5. Additional discussion of variability / heterogeneity

Spatial variability in the measured isotope compositions may arise from variations in ice thickness, irradiation dose, and surface heterogeneity. For the samples that were covered in water ice prior to irradiation, water-ice thickness variations would lead to variations in electron fluences that penetrated the surface of the samples. For the pre-irradiated samples non-uniformities in the amounts of water ice deposited locally may have led to variations in the fraction of defects that reacted with water.

S6. Supplementary figures and additional data (Experimental)

Electron irradiation experiments at T=10 K were carried out using the Isotopic Characterization Experiment (ICE) at CSU San Marcos. Electron irradiation times of ~45 minutes (low e- fluence) and ~ 450 minutes (high e- fluence) were done using an electron gun (Kimball Physics) beam current of ~50 μAmps and energies of 5 keV/electron. These fluences and energies were chosen to maximize the number of secondary electrons, beam penetration, and defects created in the solid targets. We note that while the temperature of the sample holder (See Figure S4) indicated that T=10K on the edge of the target (thermometry provide by a calibrated Si diode and Lakeshore LS-335 temperature controller), it is possible that the temperature at the center of the target may have systematically warmed up as a portion of the energy deposited by 5 keV electrons was converted to thermal energy via lattice vibrations.

In some experiments, water vapor was deposited and frozen out prior to electron irradiation and in others it was deposited after the electron irradiation was completed.

Isotope ratio determinations of blank and electron irradiation exposed Al_2O_3 (sapphire) and SiO_2 (quartz) surfaces were performed using the CAMECA IMS-1290 secondary ion mass spectrometer at UCLA.

Materials Used. Electron irradiation targets, optical quality disks 10 mm diameter and 1-3 mm thick, made of either (SiO_2-quartz) or Al_2O_3 (sapphire) were acquired from ColdEdge Technologies to fit into the optical substrate holder (See Figure S4).

Vienna **S**tandard **M**ean **O**ceanic **W**ater (VSMOW) and **G**reenland **I**ce **S**heet **P**recipitation (GISP) water standards were acquired from National Institutes of Standards and Technology (NIST). Their isotopic compositions are listed in Table S2.

Simultaneous Exposure Experiments. At the start of a new target exposure experiment, the cryocooler's 6-inch CF flange is unbolted and the cold-head from the UHV chamber is carefully withdrawn (See Figure S4). New target disks are carefully[1] placed inside a cylindrical well in the gold-plated sample holder attached to the cold-head and secured using a gold-plated cover piece, indium wire, and UHV rated screws. Once secured and inspected for fingerprints and other contamination, the cold-head and sample holder are remounted carefully and aligned to maximize electron beam fluence.

The first set of experiments that we completed were simultaneous irradiation of H_2O-ice and solid surfaces. To produce these water-ice covered surfaces we first pumped out the heated (T=40 degrees C) UHV chamber overnight until the pressure was P~10^{-9} Torr or less to minimize unwanted H_2O backgrounds acquired from venting the chamber. We prepared water ice samples by injecting 5-10 μL of a GISP or VSMOW water standard into the vacuum line followed by controlled deposition of this water onto the cold solid surface using a stainless-steel needle valve. Once the pressure inside the UHV stabilized below ~ 2×10^{-8} Torr, we initiated electron irradiation and monitored the electron flux every 10 minutes for 30 seconds using a Faraday cup, pico-Ammeter, and MATLAB data acquisition software developed at CSUSM. This monitoring is necessary as adjustments sometimes need to be made to the cathode voltage in order to maintain a steady beam current. Once electron irradiation was complete, the He compressor was turned off, and the cold surface was brought up to room temperature while simultaneously pumping the UHV chamber using the Shimadzu magnetically levitated Turbo pump (TMP-303LM). Because we wanted to minimize water-liquid-vapor interactions, we did not collect the residual H_2O for the experiments reported here. Pressures less than ~ 3×10^{-8} Torr were maintained throughout the electron bombardment, minimizing the potential for any gas-phase chemistry.

Pre-exposure Experiment. One sample (SiO_2–2) was pre-irradiated and then exposed to a total of 30 μL of GISP ($\delta^{18}O = -24.78 \pm 0.09‰$) H_2O water vapor while being held at 10 K. Once irradiations and water-ice exposures were done, samples were stored in clear plastic containers until they were analyzed at the NSF UCLA SIMS facility. See Fig. S6 for evidence of H_2 gas production.

High Fluence Experiment. The high fluence experiment (Al_2O_3-10) was conducted using similar protocols as the low-fluence experiment (SiO_2-7) using 5 microLiters of GISP water to coat the surface followed by ~450 minutes of e-beam exposure. After electron beam exposure, we did five rounds of 5 microLiters of GISP water vapor deposition while holding the surface at T = 10 K.

Control Experiments. For completeness, we carried out several experimental controls to evaluate whether we would have seen isotopic shifts in the solid substrates without the added water and/or e- irradiation. These are summarized in the Table S3 below.
The control experiments show that the anomalous oxygen isotopic results are the result of having both electron irradiation and H_2O ice present in the experiments.

[1] Using UHV standard techniques including facemasks, beard covers (when applicable), hair nets and UHV rated gloves.

SIMS Measurements (UCLA) and Standards (UCLA). The oxygen isotopic composition of irradiated and non-irradiated (standard) surfaces and near surface regions was determined using the UCLA IMS-1290 ion microprobe. The samples were raster-sputtered (50 µm x 50 µm) with a 3 nA Cs^+ primary ion beam by collaborator. Secondary ion intensities were measured in multi-collection mode under a mass resolution (M/∆M) of 6,000, with ^{16}O, ^{17}O and ^{18}O being collected with L'2 (Faraday cup, 10^{10} Ω), axial electron multiplier (EM) and H1 (Faraday cup, 10^{11} Ω), respectively. A 35 µm by 35 µm field aperture was used to ensure that only signals from the center of a crater were collected. No pre-sputtering was applied prior to data acquisition. Non-irradiated quartz and sapphire were used as standards to infer the deviation of oxygen isotopic compositions from a mass-dependent fractionation line (See Figure S5 for quartz blank).

Evidence of Hydrogen Gas Production from Pre-irradiated Sample. See Figure S6.

Figures

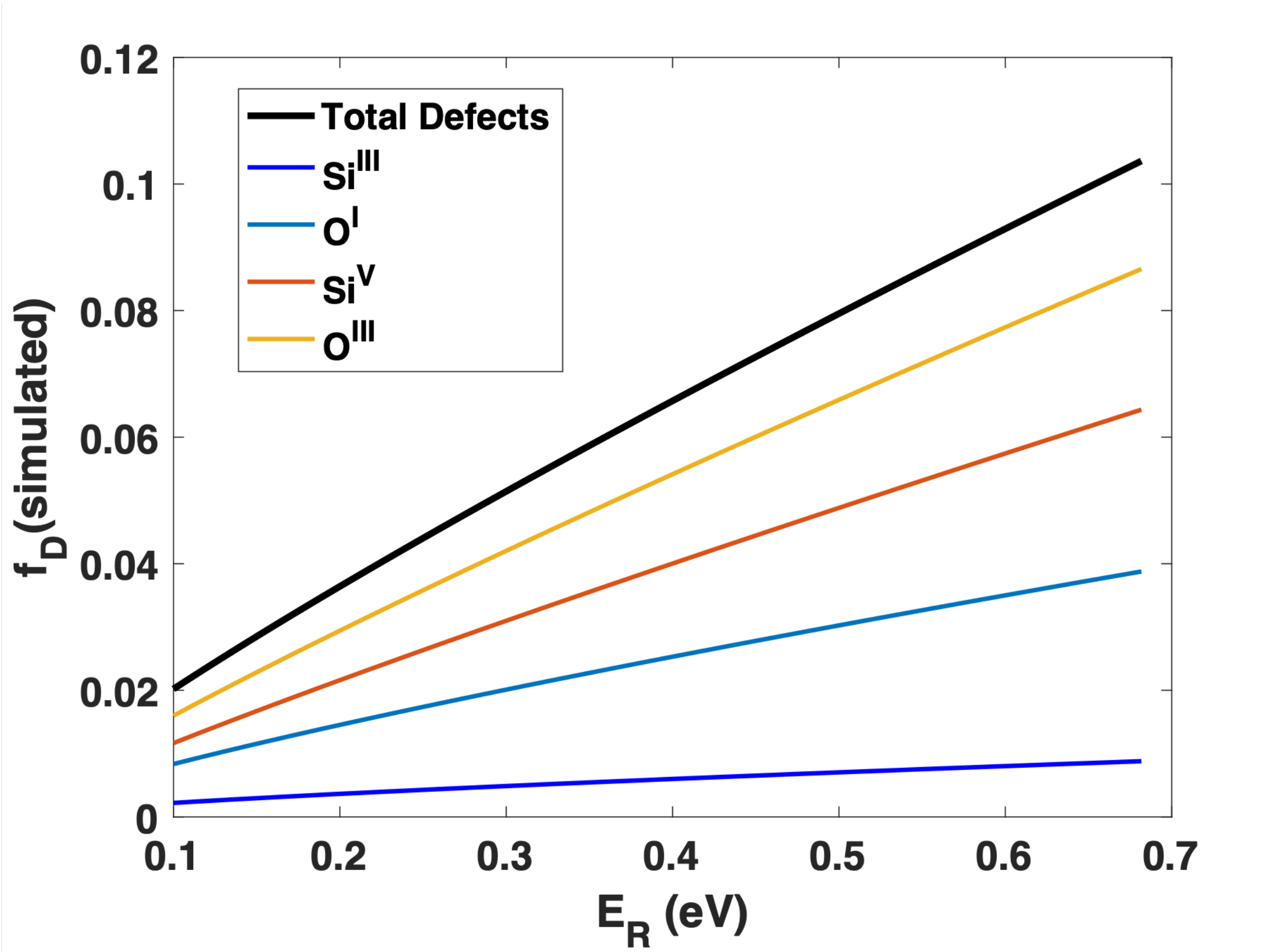

Fig S1. Maximum defect fraction (**f_D**) as estimated using the empirical relationship between simulated number of detects per PKA atom and its energy E_R ($N_d = AE_R{}^m$) where A and m are parameters derived from MD simulations of defect formation as reported by (1).

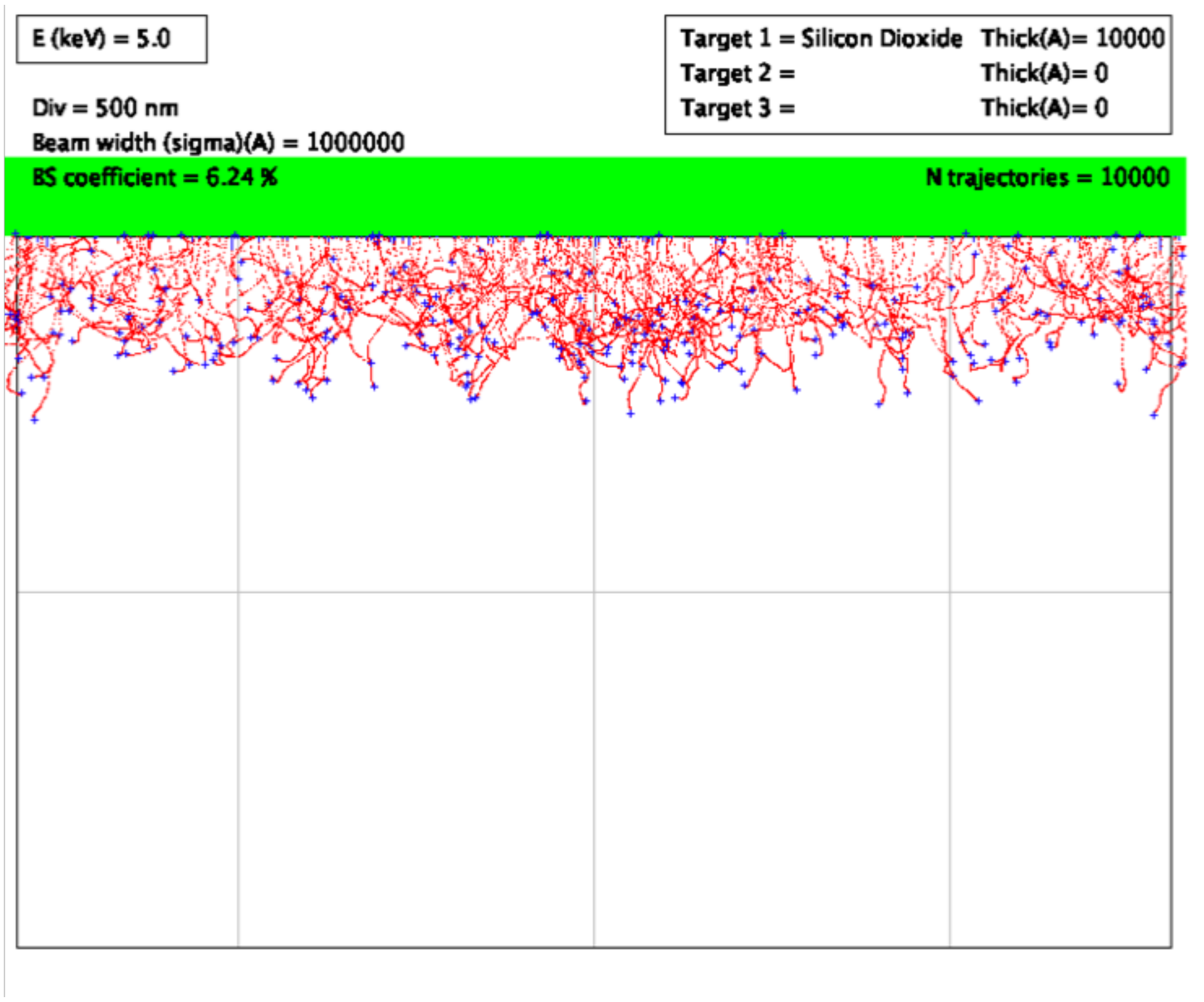

Fig. S2. Results of a single scattering simulation of 5 keV electron propagation in SiO_2. The vertical axis corresponds to penetration depth beneath the surface, and the horizontal axis represents lateral distance. We note that region heavily influenced by primary electrons is approximately ~200 nm, with blue dots indicating primary electron final location.

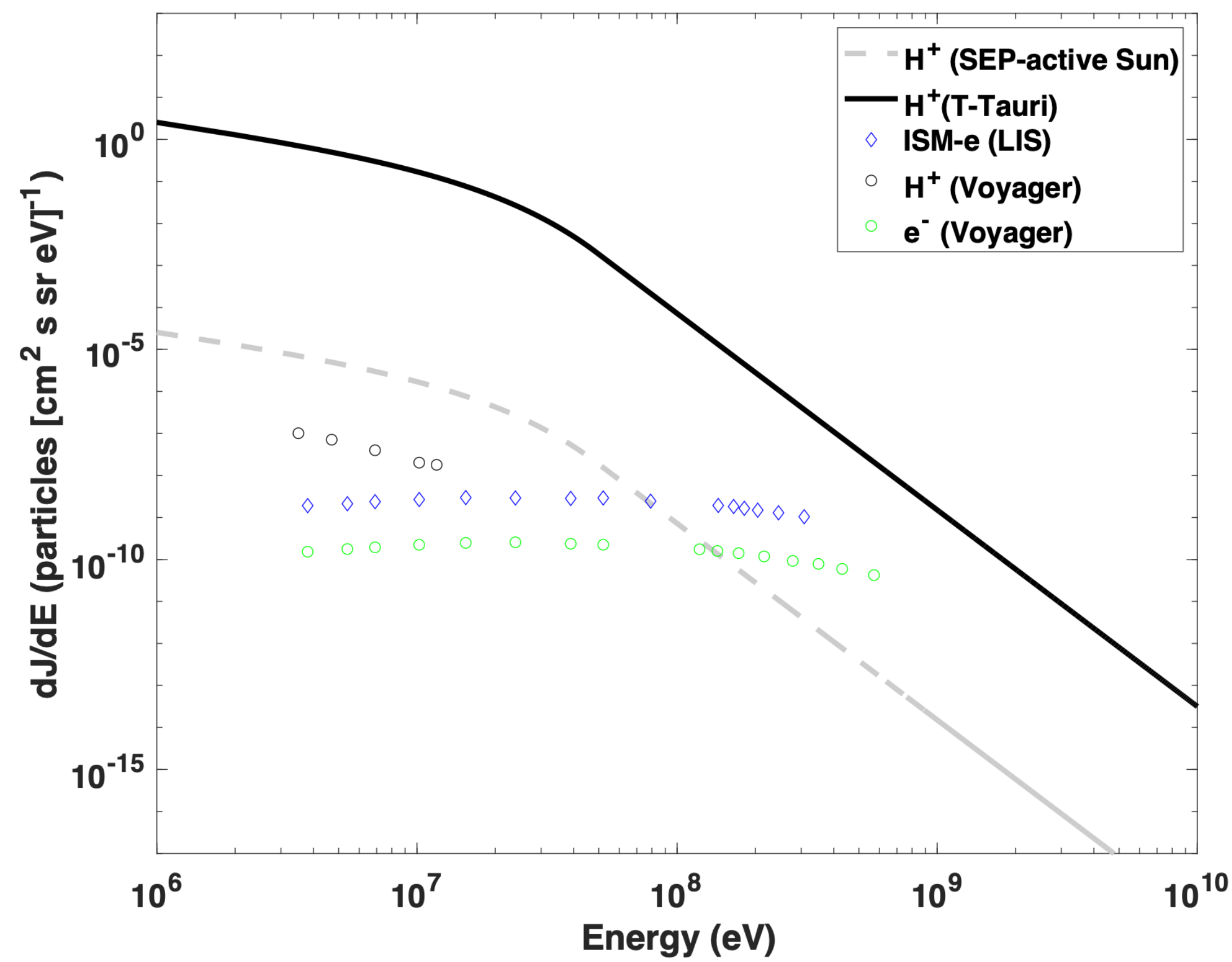

Fig. S3. Summary of cosmic ray intensity (J) spectra used for estimates of CR fluences, energy deposition, and defect formation timescales. Proton and electron cosmic ray spectral intensities as reported by Voyager (4). SEP H^+ events reported by (5) (SEP-active Sun) were scaled by a factor of 10^5 to estimate the T-Tauri H+ spectral intensities as described by (7) . For comparison, the electron component of galactic cosmic rays (ISM-e) was reproduced as described in (9).

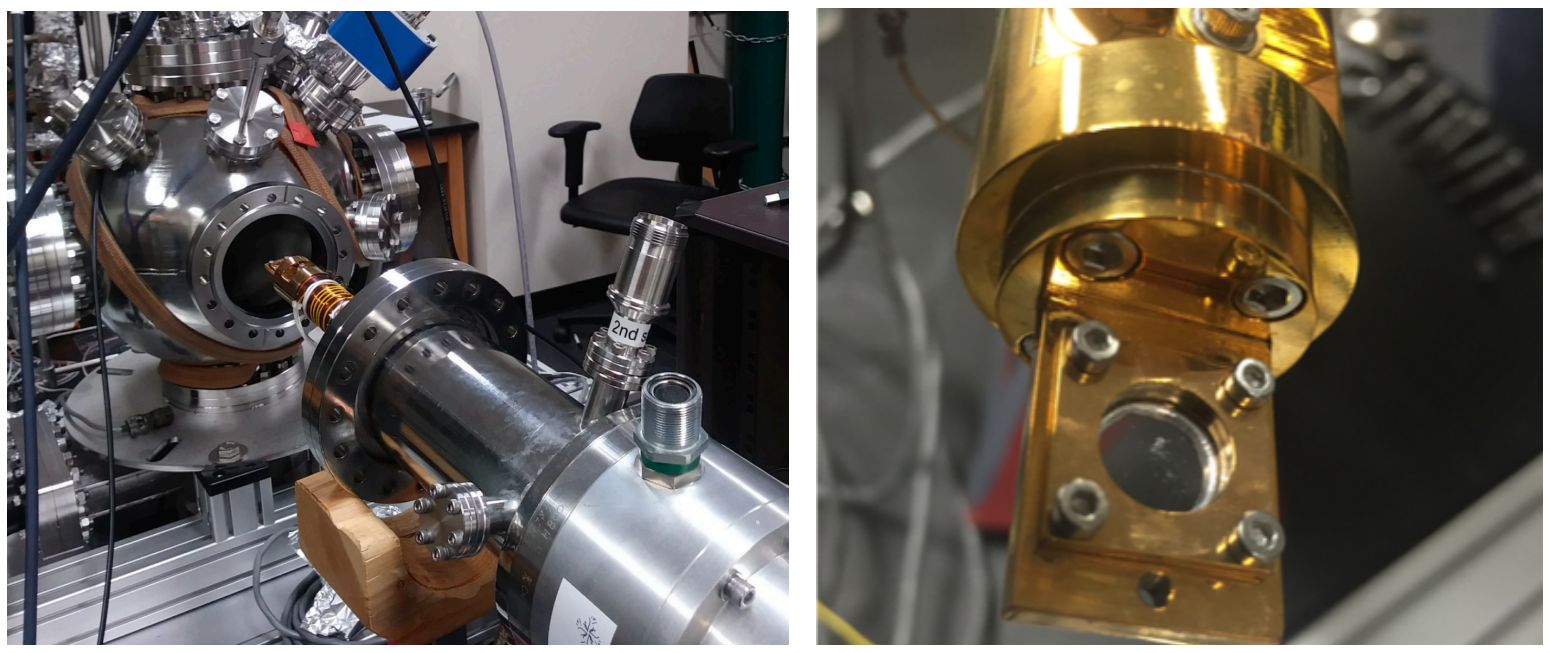

Fig. S4. (Left) View of UHV chamber and cryocooler with cold-head and sample holder. (Right) Close up of sample holder with Al_2O_3 window and mounting cover and Si diode thermistors for temperature monitoring.

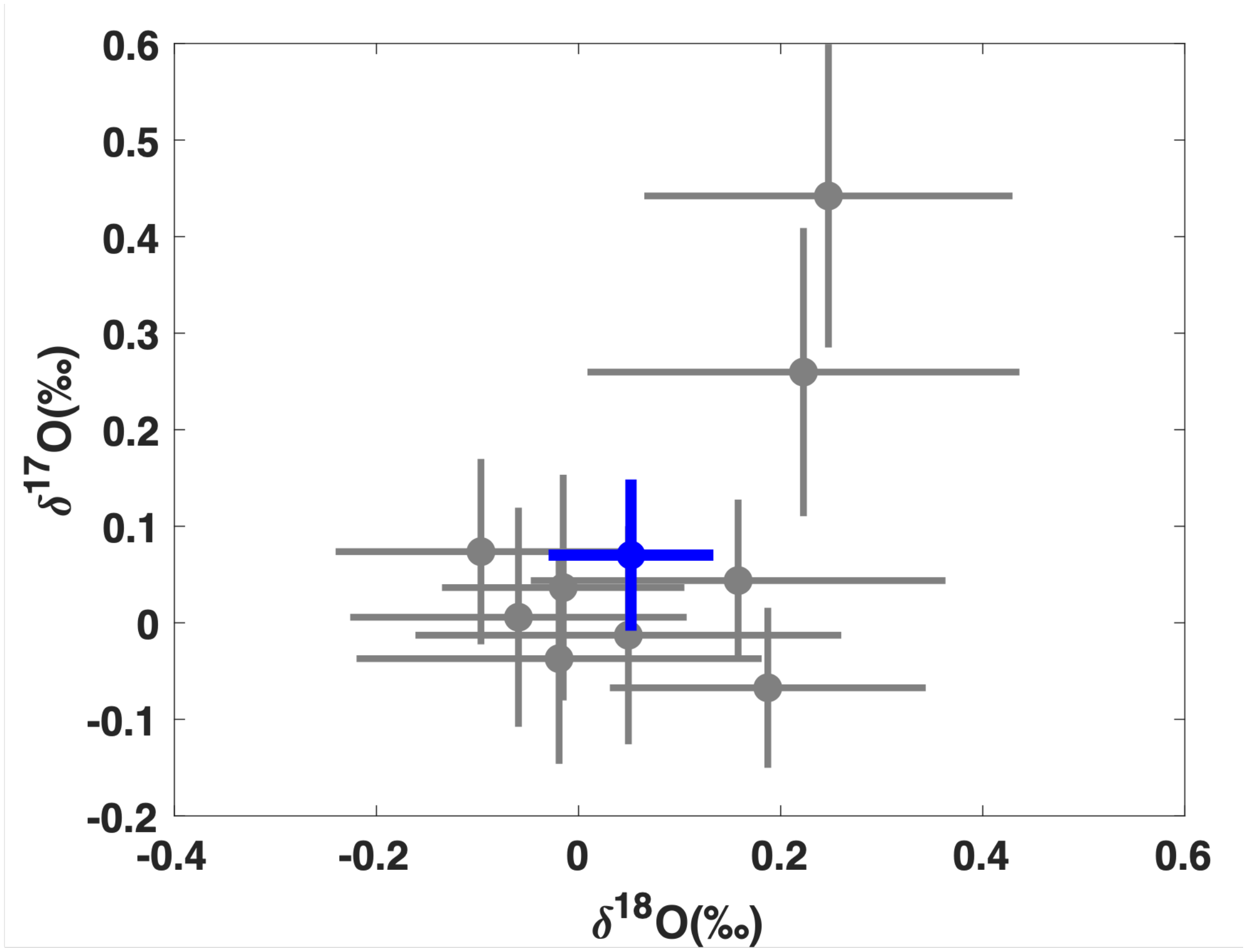

Fig. S5. SIMS analysis spots (gray) of unirradiated quartz (SiO_2) measured in delta units with respect to the weighted average of these spots (shown in blue).

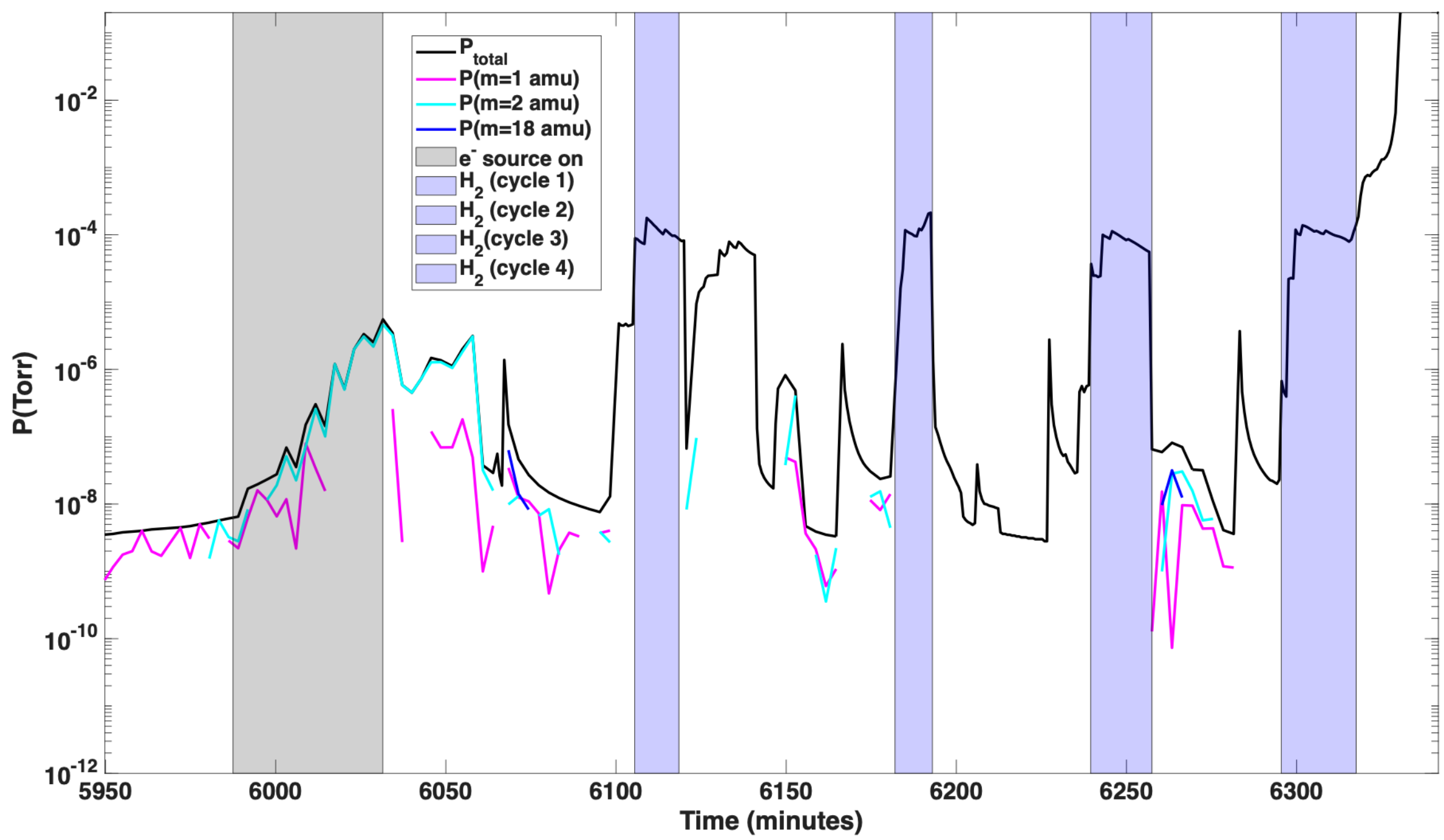

Fig. S6 A Granville Phillips Ion-trap mass spectrometer (m=1-300 amu) collected pressure and partial pressure data during experiment that produced SiO2-2 (pre) sample. Denoted is the time periods when electron source was on (gray), which appears to have produced some H_2 gas. The four cycles of 5 microliter GISP water exposure were followed by periods of time when surface of the sample was raised up to T=40K. A non-condensable gas with partial pressure of up to ~ 10^{-4} Torr was observed after each of these cycles (purple regions) when temperature of the surface exceeded 25-30K on its way up to 40K. H_2 gas was pumped out before resuming cycles.

Tables

Table S1 Summary of major variables and quantities used to calculate defect formation rates in dust grain volumes

Variable	Description	Description	Units
$S(E_{CR})$	$= \frac{dJ}{dE}$	Cosmic-ray energy Spectrum	Particles / [MeV cm^2 sr s]
Φ_p	$= 4\pi \int_{E_{min}}^{E_{max}} S(E_{CR})\, dE_{CR}$	Total Number of Particles between E_{min} and E_{max}	Particles / (cm^2 s)
$\frac{dE(E_{CR})}{dz}$	Empirical look up table (Ref. # 16)	Energy-loss per unit length of particle (H^+, e^-)	eV/cm
$E_D(E_{CR}, r_g)$	$\simeq 2r_g \frac{dE(E_{CR})}{dz}$	Amount of energy deposited in a dust grain of radius r_g	eV
$N_D(E_{CR}, r_g)$	$= F(E_D(E_{CR}, r_g))$	Number of defects created in a dust grain with radius r_g per cosmic ray particle with energy E_{CR}	Number
$\frac{dN_D(r_g, E_{min}, E_{max})}{dt}$	$= 4\pi \int_{E_{min}}^{E_{max}} N_D(E, r_g) \frac{dJ(E_{CR})}{dE} r_g^2 dE_{CR}$	Rate of defect formation in dust grain of radius r_g	Number/s
τ_D	$= \frac{N_O(r_g)}{\frac{dN_D(r_g)}{dt}}$	Oxygen isotope exchange timescale	s

Table S2. Water standards used in experimental work described below and in main text.

H_2O Standard	$\delta^{18}O$ x1000	$\delta^{17}O$ x1000	$\delta^{2}H$ x1000
**VSMOW**	0 ‰	= 0.52 x $\delta^{18}O$	0 ‰
**GISP**	−24.78 ± 0.09‰	= 0.52 x $\delta^{18}O$	-189.5 ‰

Table S3: Summary of control experiments and SIMS results.

Control # (sample)	Temp.	e^- irradiation	H_2O ice deposition	$\Delta^{17}O$ (‰)	m	R^2	Comment
1 (Qz-13)	10 K	0 min	10 mL GISP	-0.84 - 1.61 ‰	0.81	0.67	Water alone does not produce clear slope 1 effect
2 (Qz-14)	300 K	Pre, 45 min	10 mL GISP, after e^-	-0.49 – 0.88‰	0.46	0.84	Room temperature irradiation does not enhance effect or produce slope 1 effect

SI References

1. B. Wang, Y. Yu, I. Pignatelli, G. Sant, M. Bauchy, Nature of radiation-induced defects in quartz. *The Journal of Chemical Physics* **143**, 024505 (2015).
2. D. Drouin *et al.*, CASINO V2.42—A Fast and Easy-to-use Modeling Tool for Scanning Electron Microscopy and Microanalysis Users. *Scanning* **29**, 92–101 (2007).
3. M. J. Berger, Coursey, J.S., Zucker, M.A., and Chang, J. (2005) STAR, PSTAR, and ASTAR: Computer Programs for Calculating Stopping-Power and Range Tables for Electrons, Protons, and Helium Ions (version 1.2.3). (National Institute of Standards and Technology, Gaithersburg, MD).
4. A. C. Cummings *et al.*, GALACTIC COSMIC RAYS IN THE LOCAL INTERSTELLAR MEDIUM:VOYAGER 1OBSERVATIONS AND MODEL RESULTS. *The Astrophysical Journal* **831**, 18 (2016).
5. R. A. Mewaldt *et al.*, Proton, helium, and electron spectra during the large solar particle events of October–November 2003. *Journal of Geophysical Research: Space Physics* **110** (2005).
6. Anonymous, 4. Charged-Particle Stopping Powers and Related Quantities. *Journal of the International Commission on Radiation Units and Measurements* **14**, 21–30 (2016).
7. C. Rab *et al.*, Stellar energetic particle ionization in protoplanetary disks around T Tauri stars. *A&A* **603** (2017).
8. A. P. Jones, J. A. Nuth, Dust destruction in the ISM: a re-evaluation of dust lifetimes. *Astronomy and Astrophysics* **530**, A44 (2011).
9. M. S. Potgieter, E. E. Vos, R. Munini, M. Boezio, V. D. Felice, MODULATION OF GALACTIC ELECTRONS IN THE HELIOSPHERE DURING THE UNUSUAL SOLAR MINIMUM OF 2006–2009: A MODELING APPROACH. *The Astrophysical Journal* **810**, 141 (2015).
